# Disseminated peritoneal leiomyomatosis: A benign condition with diagnostic and therapeutic challenges

**DOI:** 10.5339/qmj.2024.40

**Published:** 2024-09-24

**Authors:** Olusola O. Akanbi, Paul Omoregie, Uche A. Akunaeziri, Muritala O. Wakeel, Nelson O. Iyabor, Oluwasegun A. Akanni

**Affiliations:** 1 Surgery Department, Federal Medical Centre Keffi, Keffi, Nigeria *Email: drssolaakanbi@yahoo.com; 2 Obstetrics and Gynaecology Department, Federal Medical Centre Keffi, Keffi, Nigeria; 3 Obstetrics and Gynaecology Department, Ladoke Akintola University of Technology Ogbomoso, Ogbomosho, Nigeria

**Keywords:** Disseminated peritoneal leiomyomatosis, fibroid, fibromyoma, gynaecological diagnosis, therapeutics, challenges

## Abstract

**Background:**

Disseminated peritoneal leiomyomatosis (DPL) is a variant of parasitic leiomyomas that is characterized by multiple peritoneal and subperitoneal nodules of proliferating smooth muscle cells that histologically resemble uterine leiomyoma. We report a case of recurrent DPL to highlight its diagnostic and therapeutic challenges at the Federal Medical Centre, Keffi, Nigeria.

**Case Report:**

The patient is a 25-year-old woman with a previous history of myomectomy 3 years before presentation to the hematology unit on account of abdominal lymphoma. Based on the working diagnosis, she was referred to the general surgery unit for an open biopsy and cytoreductive surgery. She was explored, and intraoperative findings were in keeping with multiple well-circumscribed intra-abdominal masses of varying sizes. The multiple and widespread locations of the masses precluded the complete removal of the masses. Four months post-surgery, she presented with similar lesions and had a repeat laparotomy. At the surgery, she had a total abdominal hysterectomy with bilateral salpingophorectomy and excision of the abdominal masses. She was then placed on letrozole, which prevented further tumor growth and abated her symptoms.

**Discussion:**

DPL is often rarely diagnosed preoperatively and thus poses a diagnostic challenge, with many cases asymptomatic and therapeutic challenges due to its tendency to recur. Its management currently lacks consensus and is often determined by many factors, such as the age of the patients, the number of nodules, and the desire to have more children, among others. Surgical excision combined with hormonal therapy is recommended for patients who wish to conceive. For postmenopausal women and those who no longer desire conception, total abdominal hysterectomy with bilateral salpingo-oophorectomy should be considered to prevent recurrence.

**Conclusion:**

DPL is a rare form of multiple extrauterine leiomyomas. We report a case of DPL in a woman that was managed with surgical intervention and hormonal manipulation therapy following the failure of the initial surgical excision alone. We thus suggest a combination of surgical intervention and postoperative hormonal manipulation in its management, as such a multi-modality of therapy was employed in the index case without evidence of recurrence after a year post-surgery.

## Introduction

Disseminated peritoneal leiomyomatosis (DPL) is a benign smooth muscle tumor that is characterized by widespread multiple peritoneal and subperitoneal nodules of varying sizes.^1^ The entity was first reported in 1952 by Wilson and Peale.^2^ Though the origin of the tumor is still debatable, the majority of the cases reported often followed uterine surgery and thus have been considered as a disseminated form of parasitic leiomyoma that was first described in 1909 by Kelly and Cullen,^3^ which is a form of extrauterine fibroid that falls into the atypical class of the International Federation of Gynecological and Obstetrics classification of fibroid.^4^ Various theories have been put forward concerning its pathogenesis, such as peritoneal metaplastic transformation,^5^ the seeding of a uterine leiomyoma fragment during myomectomy ^6^ or following spontaneous rupture of subserosa fibroid into the peritoneal cavity.^7^ Few cases of DPL have been reported, and there are no current standard guidelines for its management. We report a case of DPL in a 25-year-old woman who underwent an open myomectomy 3 years earlier to highlight the diagnostic and therapeutic challenges in managing DPL. Institution approval and signed patient consent were obtained for this case report, and associated images were used in this case report.

## Case Report

The patient is a 25-year-old gravidae 3 para 2 alive (G3P2^+1^) woman, who was referred to the general surgery unit from the hematology unit of the Federal Medical Centre, Keffi, Nigeria, for cytoreductive surgery and a biopsy of intra-abdominal masses based on a working diagnosis of abdominal lymphoma. On review, the main complaints were progressive abdominal swelling, early satiety, chronic constipation, and weight gain over 3 months before presentation. Past medical history was in keeping with a previous history of a myomectomy done about 3 years earlier that was uneventful. The review of other systems was not remarkable.

**Figure 1.**
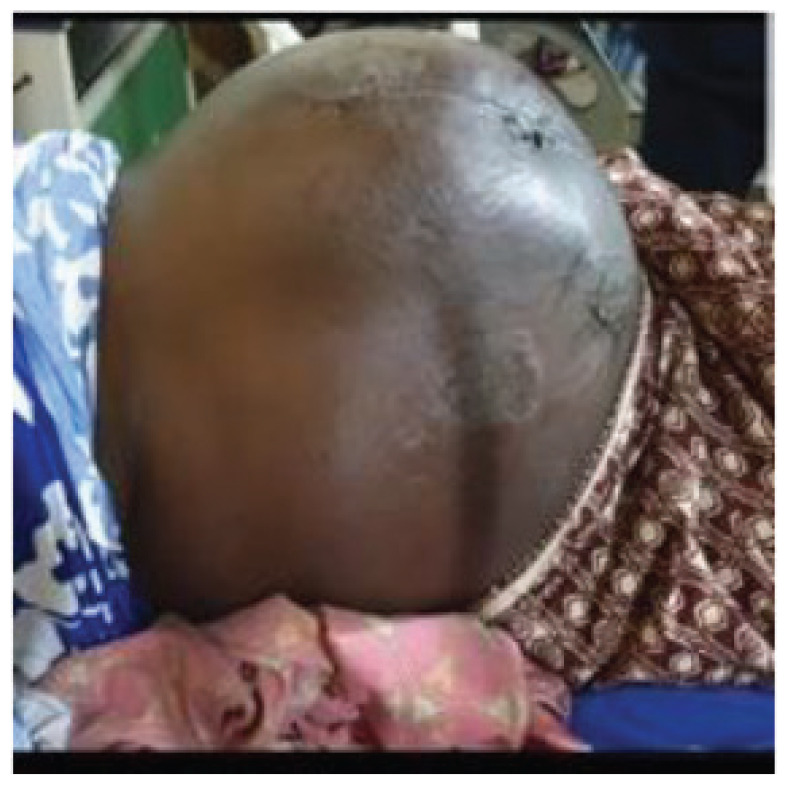
The patient’s abdomen at first presentation.

On examination, the patient was in respiratory distress and apprehensive; otherwise, the general examination was essentially normal. The abdominal findings were in keeping with gross abdominal distension with a lower sub-umbilical scar (Figure 1) and multiple palpable mobile intraabdominal masses with minimal ascites. The vaginal examination and per rectal examination were not remarkable.

Plain abdominal X-ray and abdominopelvic ultrasound were ordered, and the findings were in keeping with dilated small and large bowel loops with multiple discrete intraabdominal masses extending from the pelvis to the upper abdomen and free peritoneal fluid. Blood chemistry, complete blood count, and urinalysis were essentially within normal limits.

**Figure 2.**
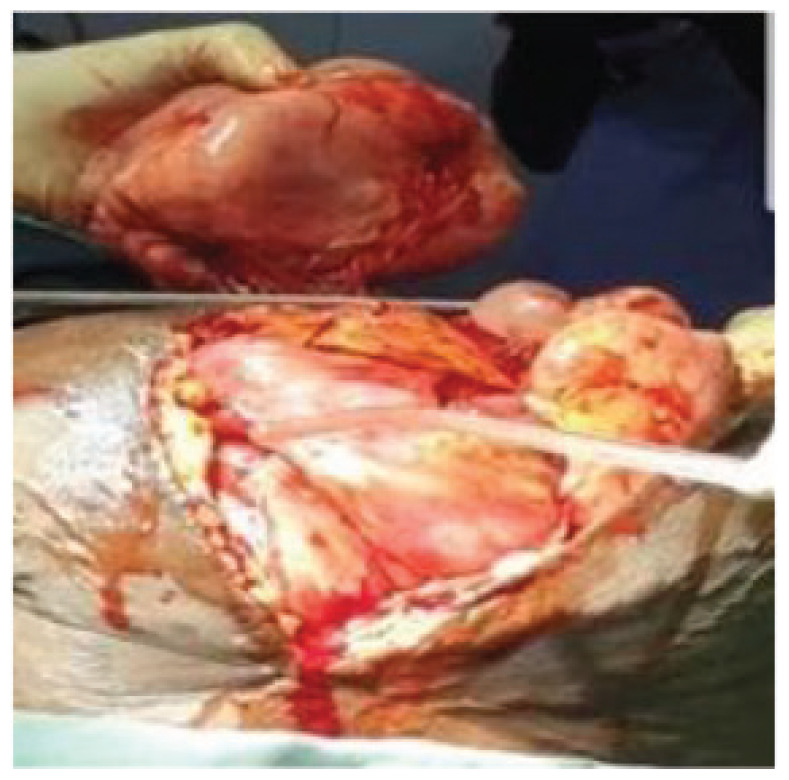
Intraoperative view at first surgery showing a mass attaching to the greater omentum.

**Figure 3.**
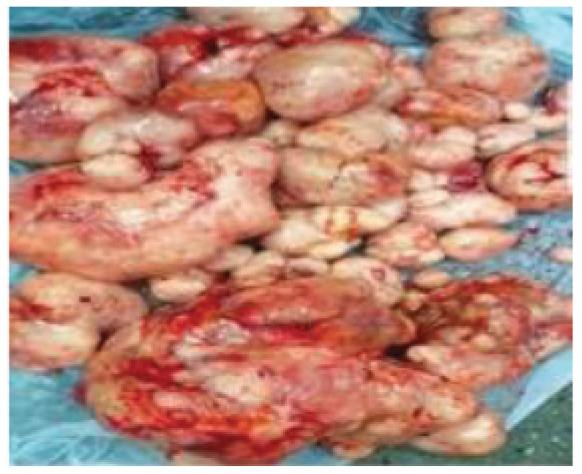
Some excised masses at first surgery.

The patient had an emergency laparotomy under general anesthesia. The intraoperative findings were in keeping with multiple discrete mobile intra-abdominal and pelvic masses of various sizes ranging from 0.5 × 0.5 cm to 20 × 25 cm. with some on the surface of the uterus, liver, bowel, and omentum (Figures 2 and 3). The ovaries were essentially grossly normal.

The patient then had enucleation of the masses and an omentectomy. Other smaller masses within the abdominal recesses, parietal peritoneum, hepatic surface, and small bowel mesentery were also excised as much as possible, with the excised masses weighing about 6.4 kg. A complete excision of the masses could not be achieved due to the multifocality of the lesion. The histology of the excised mass was in keeping with the proliferative smooth muscle tumor without evidence of malignancy. She had a total of six units of blood transfused in the postoperative period. She had an uneventful postoperative period and recovered well. She was discharged on the eighth postoperative day to be seen in the clinic but did not attend the scheduled follow-up visit and only returned after 4 months with a similar presentation.

On her second visit, a working diagnosis of Abdominal Compartmental Syndrome secondary to multiple recurrent and retained intraabdominal masses was made.

**Figure 4.**
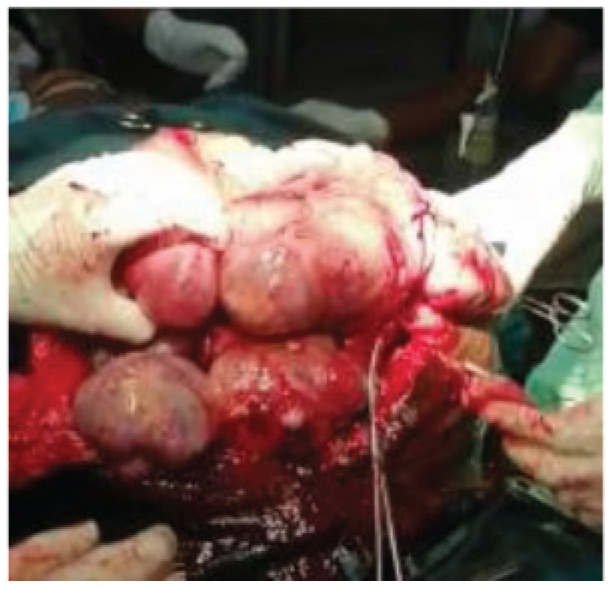
Intraoperative view at repeat laparotomy.

The patient was then thoroughly evaluated in preparation for a repeat laparotomy and counseled for a total abdominal hysterectomy with bilateral salpingo-oophorectomy. She had a repeat laparotomy in conjunction with the obstetrics and gynecology team. The intraoperative findings at the repeat laparotomy are shown in Figure 4.

At surgery, she had a resection and enucleation of the peritoneal masses, and total abdominal hysterectomy plus bilateral salpingo-oophorectomy (TAH plus BSO). She was transfused with four units of blood during the postoperative period; otherwise, the postoperative period was uneventful, and was discharged to the follow-up clinic on the 7th postoperative day. The patient was placed on 2.5 mg of letrozole daily for 12 months. At 13 months post-surgery she had no clinical or radiological evidence of recurrence, at which point she was lost to follow-up.

## Discussion

Leiomyoma is a benign uterine tumor composed mainly of smooth muscle with some connective tissue. The tumor is classified into four subclasses based on its location in relation to the uterine layer, where it is located: submucosa, intramural, subserosa, and pedunculated.

Parasitic leiomyoma is a form of extrauterine leiomyoma and is classified as type 8 leiomyoma based on the FIGO classification of fibroid.^4^

DPL is often rarely diagnosed preoperatively and thus poses a diagnostic challenge,^8^ with many cases asymptomatic and when symptomatic, it presents with features of abdominal discomfort or intraabdominal masses with features of a space-occupying lesion.^9^ This asymptomatic period was also observed in our case.

The theoretical explanation responsible for DPL includes the seeding theory, which occurs when a uterine leiomyomas fragment falls into the peritoneal cavity and gets implanted during the operative procedure of myomectomy; morcellation of the myoma during a laparoscopic procedure is also considered, as a risk factor for such dissemination and seeding of leiomyoma tissue into the peritoneal cavity, as many of the previously reported cases were seen in patients that underwent laparoscopic myomectomy.^7,10^ Another proposed theory is that of implantation of spontaneously ruptured subserosa fibroid into the peritoneal cavity, which by neovascularization, takes blood supply from extrauterine structure, mainly from the greater omentum or mesentery of the small bowel.^3^ Another theory is the hormonal-metaplastic theory, which proposes that exposure to an elevated level of female steroid induces metaplastic transformation of a mesenchymal cell of the peritoneum.^5^ This form of peritoneal leiomyomatosis (PL) is often associated with the presence of nodules in unusual locations in the abdomen^5^ and when it is multiple, it is termed leiomyomatosis peritonealis disseminate, a condition that was first described by Wilson et al.^2^

In many of the previously reported cases of PL, the majority of the nodules were often single and located in the pelvis, with few cases involving the upper abdomen. In the current case, the nodules involved the entire abdominopelvic cavity.

The management of PL and its DPL variant currently lacks consensus ^11^ and is often determined by many factors, such as the age of the patients, the number of nodules, and the desire to have more children, among others.

Though surgical excision and resection of the omentum are surgical options employed in majority of the reported cases, with all claiming complete resolution after 3 months of follow-up. This was contrary to our case, as the multiple locations of the nodules and the presence of miniature, barely visible multiple seedings covering more than 70% of the peritoneum precluded complete excision of the nodules, which predisposed our patient to recurrence after the first surgery. In such a case, the use of hormonal therapy with aromatase inhibitors, gonadotrophin-releasing hormone injections, or selective progesterone inhibitors may be of benefit, as seen in our case, in which there was no clinical or radiological evidence of recurrence in the patient after 8 months of follow-up following a repeat laparotomy. The use of hormonal therapy has been previously suggested as one of the options of treatment for the prevention of recurrence, and in patients who desire to have more children,^12,13^ while for postmenopausal women and women who no longer desire conception, TAH plus BSO should be considered as an alternative treatment option to prevent a recurrence.

## Conclusion

In conclusion, DPL is a rare variant of parasitic leucomyoma and often develops following uterine surgery, and its diagnosis should be entertained in women with multiple intra-abdominal masses following a previous history of uterine surgery. Surgery remains the main therapeutic option in symptomatic cases, and when complete resection is impossible, as seen in our case, hormonal manipulation should be considered earlier and not until recurrence occurs. We thus recommend copious lavage of the peritoneal cavity as this may minimize the myoma tissue left in the peritoneal cavity after open or laparoscopic myomectomy and when faced with the diagnosis, a combination of surgery with early hormonal therapy is advocated. We also suggest a need for more research into the etiopathogenesis of DPL, as many of the previously identified factors may just be co-factors in the development of DPL.

## Authors’ Contributions

Literature search: OA, UA, MW, NI, OAA. Manuscript preparation: OA, PO, UA. All authors have read, reviewed, and approved the final version of manuscript.

## Consent

The authors declared that the patient’s consent forms were obtained. In the form, the patient was informed that his image and clinical details would be reported and published if accepted by a peer-reviewed journal. The patient was also informed that there would be no mention of his name or initial and all efforts would be made to conceal her identity, but complete anonymity cannot be guaranteed.

## Conflict of Interest Statement

The authors declare that they have no competing interests.
